# Metabolic Effects of Testosterone Hormone Therapy in Normal and Orchiectomized Male Rats: From Indirect Calorimetry to Lipolytic Enzymes

**DOI:** 10.1155/2019/7546385

**Published:** 2019-11-28

**Authors:** Mahmoud Mustafa Ali Abulmeaty, Ali Madi Almajwal, Mohamed Farouk ElSadek, Mohamed Y Berika, Suhail Razak

**Affiliations:** ^1^Department of Community Health Sciences, College of Applied Medical Sciences, King Saud University, Riyadh, Saudi Arabia; ^2^Medical Physiology Department, School of Medicine, Zagazig University, Zagazig, Egypt; ^3^Nutrition and Food Science Department, Faculty of Home Economics, Helwan University, Helwan, Egypt; ^4^Rehabilitation Sciences Department, College of Applied Medical Sciences, King Saud University, Riyadh, Saudi Arabia; ^5^Faculty of Medicine, Mansoura University, Mansoura, Egypt

## Abstract

**Background and Aim:**

Changes in total energy expenditure (TEE) and substrate metabolism may help explain the metabolic actions of testosterone (T). This study measured respiratory quotient (RQ), TEE, ghrelin, insulin, and key lipolysis enzyme concentrations in relation to body weight (wt) and food intake (FI) in both normal and bilaterally orchiectomized rats with/without T treatment.

**Methods:**

In total, thirty-two male Wistar rats (300–400 g) were divided into four groups (*n* = 8/group), including (a) sham-operated and vehicle-injected group (Sham), (b) T-treated sham group (T-Sham) for which sham-operated rats were injected with IM testosterone undecanoate (100 mg/kg, for one week), (c) orchiectomy and vehicle-injected group (Orch), and (d) T-replaced orchiectomy group (T-Orch). After one week, FI and wt were automatically recorded, indirect calorimetry parameters were measured, and blood samples were collected to measure T, ghrelin, insulin, growth hormone (GH), glucose, hormone-sensitive lipase (HSL), adipocyte triglyceride lipase (ATGL), free fatty acids (FFA), and lipid profiles.

**Results:**

Orchiectomy decreased ghrelin, GH, and insulin levels, increased TEE and RQ, and lowered FI and wt. The T-Orch group exhibited increased levels of ghrelin (3-fold), insulin, GH, blood levels of lipolysis products, TEE, and FI in addition to reduced glucose levels (*P* < 0.05). This group demonstrated no significant changes in wt. In the T-Sham group, T increased ghrelin and insulin levels (*P* < 0.05) with strong positive correlations (*r* = 0.663 and 0.644, respectively, *P* < 0.05), increased ATGL levels, RQ toward carbohydrate utilization ranges, and TEE, and reduced HSL levels (*P* < 0.05) with insignificant changes in FI or wt.

**Conclusions:**

T administration in orchiectomized rats significantly increased orexigenic mediators such as ghrelin and insulin without inducing any significant changes in wt. The mechanism for this finding might be the increased TEE and the stimulation of lipolysis through the ATGL enzyme. The associated rise of GH might help in interference with accumulation of lipid in adipose tissue. Apart from the effect on GH, T-Sham showed similar effects of T supplementation.

## 1. Introduction

Males with hypogonadism have distinctive metabolic derangements that mandate testosterone hormone replacement therapy (TRT), which indicates that testosterone (T) is a metabolic hormone with essential roles in substrate metabolism and energy homeostasis [1]. Being an anabolic hormone, it has also been investigated for use in healthy normal older men for improvement and maintenance of physical function and muscle mass [2]. The mechanisms of the stimulatory effect of T on feeding are still under investigation. Iwasa et al. [3] reported that the orexigenic effect of T is not due to raised neuropeptide Y (NPY) levels or any effects on leptin. In contrast, Jafaripour et al. [4] showed that T increases food intake (FI) by increasing NPY and ghrelin levels. It has also been reported that T decreases preproghrelin mRNA expression in the hypothalamus and increases expression in the gut [5]. This orexigenic effect of T is not always accompanied by weight (wt) gain in either animal research models or clinical practice. Additionally, T deficiency, at least in part, may lead to clinical conditions such as obesity, type 2 diabetes, and metabolic syndrome [1]. Taken together, it can be concluded that T increases FI without increasing body wt. This apparent paradox requires further clarification.

The study of energy expenditure (EE) is an essential part of metabolic research, especially with the availability of new equipment that can accurately measure the total energy expenditure (TEE) or its components, such as the resting energy expenditure (REE), thermic effect of feeding (TEF), and thermic effect of activity (TEA). Indirect calorimetry is a gold standard for measuring TEE in humans and experimental animals [6]. Typically, new devices are based on open-circuit indirect calorimetry in which the gas is withdrawn from the atmosphere by a pump, passed through silica gel to be dried and measured, and then regulated by a flow controller before entering the box containing the animal. The box also has an outflow port with the outflow gases being dried again to remove any water vapor produced by the animal. After drying, the outflow gases are analyzed for O_2_ volumes (VO_2_ in liters) and CO_2_ volumes (VCO_2_ in liters) [7]. Both O_2_ consumption and CO_2_ production are used to calculate the respiratory quotient (RQ). The RQ, coupled with the amount of nitrogen excretion, enables the calculation of the relative consumption of macronutrients, especially carbohydrates and fat [8]. The TEE can be calculated using the Weir equation in which heat production_(kcal/day)_ = 1.44 × [(3.94 × VO_2_ consumed) + (1.1 × VCO_2_ produced)] [9].

Lipolysis is a pivotal process in metabolism and energy homeostasis. The regulation of lipolysis is mediated via three key enzymes, hormone-sensitive lipase (HSL), the recently discovered adipose triglyceride lipase (ATGL), and monoglyceride lipase (MGL), with the main lipase activity in adipose tissue and skeletal muscle being mediated by HSL and ATGL [10]. The mechanisms by which T affects lipolysis and consequently modulates body composition and wt remain unclear. Some investigators have reported that T reduces ATGL and HSL [11], while others have reported that T increases ATGL and HSL expression [12], and yet others found no significant effect of castration or T supplementation on these enzymes [13]. Moreover, Zang et al. [14] concluded that T reduces the expression of HSL while phosphodiesterase-3B expression is increased. Changes in EE, orexigenic mediators, and fat hydrolyzers may provide a reliable explanation for the metabolic actions of T in normal and hypogonadism conditions. Based on the findings described above, the current study aimed to measure the indirect calorimetry parameters (VO_2_, VCO_2_, RQ, and TEE), ghrelin and insulin levels, ATGL and HSL enzymes, and substrate concentrations (glucose, lipid panels, and free fatty acids) in the blood of both normal and orchiectomized rats with or without T treatment in relation to changes in FI and body wt.

## 2. Methods

### 2.1. Animals and Procedures

A total of 32 male Wistar rats (300–400 g, 10–12 weeks old) were obtained in January 2018 from the animal research center in the College of Pharmacy, King Saud University, to be used in this study. The rats were hygienically reared in the animal vivarium at 25°C room temperature and 20 to 45% humidity where the lights were on from 6 am to 6 pm daily [15]. The rats were fed a dietary formulation of protein (18.1%), fat (7.1%), carbohydrate (59.3%), and fiber (15.5%) with food and water being provided ad libitum. Rats were housed in groups of four, and after one week of habituation, they were divided into four groups (*n* = 8/group), including (a) the sham-operated group (Sham), which underwent a sham surgery and received intramuscular (IM) injections of normal saline (0.3 ml) and served as normal controls; (b) the T-treated Sham group (T-Sham) in which sham-operated rats received IM injections of a preparation of testosterone undecanoate (100 mg/kg) [16] daily for one week [17]; (c) the orchiectomy group (Orch) in which orchiectomized rats received IM injections of an equal volume of the vehicle (saline); and (d) the T-replaced orchiectomy group (T-Orch) in which orchiectomized rats received TRT with daily IM injections of T at the same dose and for the same duration as the T-Orch group. The body wt (g) of all the rats was recorded before treatment, after treatment, and also during and after indirect calorimetry analysis. The study protocol was approved by the ethics committee in the College of Applied Medical Sciences, King Saud University.

### 2.2. Surgery

General anesthesia was provided via intraperitoneal injection of a ketamine/xylazine mixture (75/2.5 mg/kg, respectively) [18]. Anesthetized animals were secured in a sterile operating theatre in a supine position. The scrotal skin was then disinfected with povidone-iodine and an eye ointment was applied to the eyes. For the Orch and T-Orch groups, a linear incision in the ventral skin of the scrotum was made, dissection of subcutaneous tissues was performed, and the tunicae were opened. The spermatic cord and main blood vessels were isolated and ligated with two adjacent ligations. The lower end of the spermatic cord was then cut between the two ligations to enable bilateral removal of the testicle and epididymis. The testicular skin was continuously sutured and a local antibiotic ointment was applied over the sutures [19]. The Sham and T-Sham groups of rats underwent midline linear incisions in the ventral skin of the scrotum and dissection of the subcutaneous tissues to reveal the testes, followed by continuous suture of the skin. To ensure the metabolic and endocrinal derangements induced by this procedure, the rats were left undisturbed for two weeks after the orchiectomy [20], prior to the study protocols being applied. In the end, rats were re-anesthetized and blood was collected via cardiac puncture and the visceral fat depots (epididymal, retroperitoneal, and mesenteric) were dissected and weighed.

### 2.3. Indirect Calorimetry

After treatment week, rats of all the groups were individually housed in Calo-cages of a TSE PhenoMaster system (TSE, Germany) at 25°C. The animals were left undisturbed for 24 hours before making the calorimetry measurements to allow for acclimatization to the individual housing conditions and to minimize the so-called novelty effect [21]. After gas calibration and calibration of the food sensors, the experimental protocol was executed for three days. The volumes of respiratory gases oxygen (VO_2_) and carbon dioxide (VCO_2_) were measured based on open-circuit indirect calorimetry. In addition, the RQ and EE per hour and per kg of body wt (Kcal/h/kg) were measured. FI was automatically recorded using a calibrated sensor at a sensitivity of 0.01 g. The measurements were taken and recorded every 15 min and those of the first six hours were omitted to ensure the stability of the recording process. Data used for the analyses included VO_2_, VCO_2_, RQ, TEE, FI, and body wt [22].

### 2.4. Blood Samples

After indirect calorimetry, all rats were fasted overnight and then euthanized and approximately 7 mL of blood was collected via cardiac puncture. The blood was collected in heparinized mini collection tubes and centrifuged, and the plasma was collected and stored at −80°C until the time of analysis.

### 2.5. Hormone, Enzyme, and Substrate Analysis

Measurement of total testosterone levels was done using enzyme-linked immunosorbent assay (ELISA) kits for T (catalog number MBS262661; Mybiosource, USA) as described by Njoroge et al. [23]. Based on the information from the manufacturer, the sensitivity of the T ELISA kits was 0.05 ng/ml. A total ghrelin ELISA kit (catalog number MBS731169; Mybiosource, USA) with sensitivity of 100 pg/ml was used to measure ghrelin levels according to Ali et al. [24]. Furthermore, an insulin ELISA kit for rats with a sensitivity of 0.5 mIU/L (MBS2602037; Mybiosource, USA) was used to measure the fasting insulin levels. Growth hormone (GH) levels in plasma were measured by ELISA kits (catalog number MBS2700019; Mybiosource, USA) with a sensitivity of <49.3 pg/ml. The plasma glucose concentrations and lipid panels including triglycerides (TG), total cholesterol (TC), and high-density lipoprotein cholesterol (HDL-C) were assessed using automatic biochemical analyzers. [25]. Low-density lipoprotein cholesterol (LDL-C) levels were calculated according to the simple but accurate modified Friedewald formula [26] with the equation LDL-C = 3/4 × (TC − HDL-C), while very low-density lipoprotein (VLDL) levels were calculated by as 20% of the TG levels [27]. The insulin resistance indicated by the homeostasis model assessment-insulin resistance (HOMA-IR) was calculated according to Matthews et al. {fasting insulin (mIU/l) × fasting glucose (mmol/l)/22.5} [28]. ATGL and HSL were assessed using specific ELISA kits (catalog numbers MBS2503928 and MBS762158, respectively; Mybiosource, USA) with sensitivities of 0.469 ng/mL and <2 pg/ml, respectively. The free fatty acid (FFA) levels were measured using an FFA quantification colorimetric assay kit (catalog number MB S841629; Mybiosource, USA). The fatty acids composed of >8 carbons were quantified by either colorimetric spectrophotometry (lambda = 570 nm) or fluorometric (excitation/emission = 535/587 nm) methods with detection limits of 56.338 ng/dl of FFA. Although being an adipose tissue bound enzymes, the measurement of these enzymes in plasma is also valid [29].

### 2.6. Statistical Analysis

SPSS, version 24 for Windows software (SPSS Inc., Chicago, IL, USA), was used for all statistical analyses. The data were presented as the mean ± standard deviation (SD). Analysis of variance (ANOVA) with a post hoc test was used to analyze the differences in multiple comparisons. Pearson correlation coefficient was used to test the relationships among the study variables. *P* values <0.05 were considered to be statistically significant.

## 3. Results

### 3.1. Metabolic Characteristics of Castration as a Rat Model of Andropause

**Figure 1:**
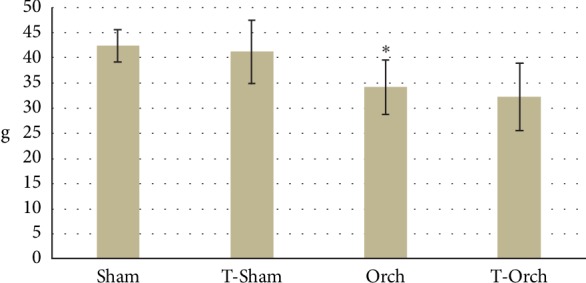
Weights of the visceral fat depots among all studied groups. ^∗^Significant versus control (Sham) group.

T levels in castrated rats were severely reduced in the Orch group compared to those in the normal control group, as well as decreased ghrelin, decreased insulin, decreased HOMA-IR, and GH levels. Hyperglycemia and reductions in lipid panels including low cholesterol, LDL, VLDL, and TG were also evident (Table 1). The Orch group also demonstrated higher EE, VO_2_, VCO_2_, and RQ in addition to lower FI, lower body wt (Table 2), and lower visceral fat depots (Figure 1). These metabolic stigmata were corrected by TRT, except for TEE, VO_2_, VCO_2_, and RQ, which were further increased (Table 2).

### 3.2. Effect of T on Ghrelin and Insulin Levels, Lipolytic Enzymes, and Substrates' Concentrations

T treatment significantly (*P* < 0.001) increased T levels in normal rats (2 fold) and restored T levels in orchiectomized rats to low points within the normal range. Ghrelin and insulin levels were significantly higher in the T-Sham group compared to those in the sham rats (*P* < 0.001) and in the T-Orch group compared to those in the orchiectomized rats (*P* < 0.001 for ghrelin and *P* < 0.05 for insulin). In addition, there was a significant reduction of ghrelin, insulin, and HOMA-IR values in the Orch group compared to those in the Sham group (*P* < 0.001). Regarding glucose, significant hyperglycemia (*P* < 0.05) was detected in the Orch group, which was normalized by TRT in the T-Orch group. All components of the lipid panel were significantly higher in the Sham-treated group compared to those in normal rats (*P* < 0.001), while in castrated rats there were significant reductions in the levels of LDL, VLDL, and TG and a significant increase in HDL. Components of the lipid profile, except HDL, increased after TRT to levels even higher than those observed in normal rats. Orchiectomy produced insignificant changes regarding HSL and FFA in adipose tissue, while ATGL showed a significant positive correlation with T levels (*r* = 0.811, *P* < 0.5). T treatment in both orchiectomized and sham-operated rats led to the significant increases in ATGL levels (3.4-fold and 4.7-fold, respectively) in addition to reduced HSL and FFA levels.

### 3.3. Indirect Calorimetry and Changes in FI and Body Weight

**Figure 2:**
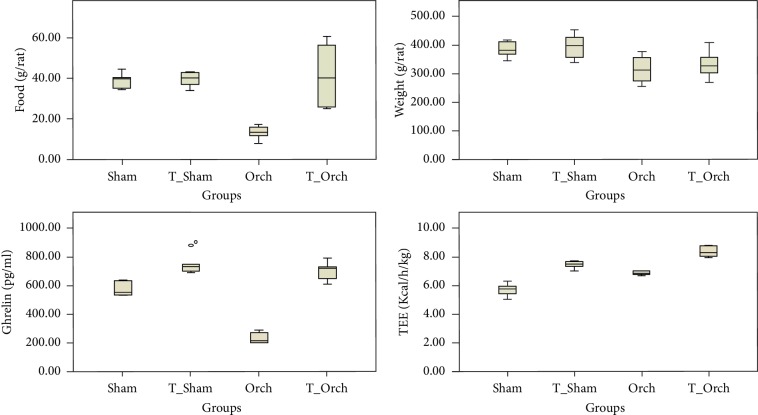
Boxplots of the food intake, weight (wt), ghrelin, and total energy expenditure (TEE) among all studied groups.

T-treated normal rats recorded significantly higher VO_2_ and much higher VCO_2_ and hence the RQ increased in comparison with that in normal control rats. Rats in the T-Orch group showed similar significant increases in both VO_2_ and VCO_2_ but led to an insignificant change in RQ in comparison with the Orch group (Table 2). T injections increased TEE in normal and orchiectomized rats, and TEE was significantly increased in the T-Sham and T-Orch groups (*P* < 0.001). Interestingly, castrated rats and those in the nontreated group also had increased TEE in comparison with the Sham group (*P* < 0.001). FI and body wt significantly decreased by orchiectomy, and T treatment failed to increase either of them (T-Sham versus Sham; *P* < 0.05). However, the T-Orch group showed significant improvement in FI with insignificant wt changes compared with that in the Orch group (Figure 2).

### 3.4. Association of T Levels with Other Study Parameters

As shown in Table 3, T levels had a strong positive correlation with ghrelin levels in the T-Sham group (*r* = 0.663, *P* < 0.5) and a strong negative correlation with ghrelin levels in orchiectomized rats (*r* = 0.754, *P* < 0.5). In normal control rats, T levels had a positive correlation with both insulin and glucose levels. A positive correlation also occurred in the T-Sham group in regard to insulin and in the T-Orch group in regard to glucose. Furthermore, T levels also showed a strong positive correlation with TEE in the T-Orch group (*r* = 0.858, *P* < 0.5), while FI showed an only moderate correlation with T levels in the Orch group (*r* = 0.584, *P* < 0.5). T treatment showed a positive correlation with FFA levels in only sham-operated rats (*r* = 0.715, *P* < 0.05). Growth hormone failed to show any significant correlations with T in all groups.

## 4. Discussion

Andropause and male hypogonadism are biochemical syndromes characterized by a deficiency of T with variable degrees of reduced sensitivity to androgen, which adversely affects many organs and deteriorates the quality of life. Energy homeostasis and body composition are affected by andropause and during TRT to variable degrees [30]. The current study measured EE and lipolytic enzymes under various androgenic conditions and correlated the parameters of indirect calorimetry with some adipokines and substrates.

Increased TEE in castrated rats was consistent with the short-term results reported by Wei et al. [31], which included increased TEE in cats nine days after castration, a postcastration period similar to that in the current study. However, Christoffersen et al. [32] reported insignificant changes in EE between castrated rats and normal controls after two weeks of orchiectomy. The results of Christoffersen and his colleagues regarding glucose and lipids were more consistent with our data. Moreover, Xia et al. [33] reported that castration-induced T deficiency resulted in an increase in glucose levels and a reduction in insulin sensitivity. It is evident that, in Orch group, the insulin level was decreased leading to higher glucose level while HOMA-IR was significantly lower than the control group, i.e., more sensitive to insulin. However, the reduced insulin level is more potent on the glucose level. The reduced GH level in castrated rats might be the cause of low HOMA-IR (Table 1). This is consistent with the finding of Sharma et al. [34, 35] who proved that GH stimulate STAT5- and MEK/ERK-dependent signaling pathways leading to change in the expression of the fat-specific protein 27 (FSP27) and peroxisome proliferator-activated receptor gamma activity (PPAR*γ*) on its promoter. As a result, GH could induce insulin resistance and lipolysis in cultured human adipocytes. Ghrelin levels decreased in one study using castrated rats [36] but increased in another study that used castrated cats [31]. The increased EE and reduced FI in orchiectomized rats produced a negative energy balance resulting in significant wt loss in the Orch group. The mechanisms of increased TEE in orchiectomized rats might include increased ATGL levels (Table 1). Haemmerle et al. [37] reported that ATGL-knockout mice have decreased the ability to acclimatize to cold exposure. Furthermore, a reduction in ghrelin itself is able to increase EE and decrease FI [38]. De Smet et al. [39] reported that young ghrelin-null mice recorded higher whole-body EE. Conversely, systemic administration of ghrelin precursor is able to reduce RQ in mice, indicating a reduced usage of lipids as a fuel substrate [40]. Another possible mechanism of the increased TEE is the evident low level of GH in the rats of Orch group (Table 1) as a result of low ghrelin. GH is responsible for tissue maintenance in adult organisms. Furthermore, it has been reported that decreased GH signaling is associated with altered profiles of adipokines, enhanced insulin sensitivity, and increased VO_2_ and TEE, which may delay the aging process [41].

Short-term intramuscular injection of T undecanoate (100 mg/kg) succeeded in raising T levels to the normal range reported by Rai et al. [42], but not to the same levels of those of the Sham group. The increase in blood lipids, such as total cholesterol, LDL, VLDL, and TG, after TRT was consistent with the results of Host et al. [43] who produced an acute TRT for chemically castrated men. In that study, the patients received gonadotropin-releasing hormone antagonist for thirty days in a randomized double-blind trial and they found that T independently increased VLDL-TG production. On the other hand, Wen and Kang [44] reported that T-treated groups at two different doses demonstrated lower concentrations of total cholesterol and LDL compared to those of castrated controls. Moreover, in that study, they also reported insignificant changes in insulin and glucose levels. Regarding the lipolytic enzymes, it may be concluded that despite the antilipolytic effects of increased insulin/ghrelin and HSL-dependent lipolytic effect of GH [45], T treatment independently induced lipolysis through increasing the levels of ATGL. However, HSL and FFA were decreased, which may have been due to increased levels of insulin [46] and ghrelin [47]. The mechanism of action of T on fat-cell lipolysis includes T effects on catecholamine signaling in adipocytes in which T increases *β*-adrenergic receptor-mediated transduction signals of lipolysis [48]. Furthermore, T increases ATGL mRNA expression and activity via the activation of PPAR*γ*^2^ receptors [12]. Overexpression of ATGL enzyme is able to reduce lipid droplet size and TG storage in adipose tissue [49]. The associated rise of GH might get rid of the accumulation of lipid in adipocytes. Zhang et al. [50] proved that in vivo and in vitro GH treatment of adipocyte cell culture enhances uncoupling protein-1 (UCP1) and adiponectin mRNA expression leading to negative effect on accumulation of lipid in adipose tissue cells.

In the current study, TRT increased TEE, VO_2_, and VCO_2_ and showed a significant positive correlation with TEE (Table 3) while having an insignificant effect on changes in body wt or mass of fat depots (Figure 2). This finding was consistent with Bauman et al. [51], who reported that TRT, even in chronic use, produces increased TEE and insignificant weight changes in hypogonadal patients with spinal cord injury. Conversely, Santosa et al. [52] reported that short-term repletion of T does not cause any changes in RQ or resting EE. Brown adipose tissue (BAT) is a logical basis for T-induced thermogenesis; however, the common mediator of UCP1, which is the primary thermogenic effector in BAT, is not directly associated with T-induced thermogenesis [53]. Santillo et al. [53] found a significantly low level of UCP-3 at one month after castration and that T administration prevented that reduction. Furthermore, T-induced thermogenesis mechanisms include the modulation of catecholamine-induced lipolysis [48] and increased levels of ATGL enzyme.

Regarding the rise of ghrelin and its positive correlation with T levels in the T-Sham group, it has been reported that T levels positively correlate with ghrelin levels in rats [34], men, and postmenopausal women [54]. Furthermore, total ghrelin levels decrease with the age of men and with declined serum testosterone levels [55]. However, in rats with a negative energy balance, a significant negative correlation exists between serum T and ghrelin [56]. Although the administration of T increased ghrelin and insulin in sham-operated rats, FI and body wt did not significantly change, indicating that T failed to increase the appetite and body wt of the rats. Skarra et al. [57] reported that, in a letrozole-induced polycystic ovary rat model, wt gain occurred secondarily to increased insulin levels and not directly to a hyperandrogenemia state. Moreover, Iwasa et al. [58] found that T-induced facilitative actions on body wt, FI, and adiposity are controlled by the coadministration of estradiol. In other words, T has inhibitory effects on wt gain and FI in the absence of estradiol supplementation. Recently, it was reported that neonatal testosterone helps in the programming of sexual differences in the hypothalamic-pituitary-gonadal-orexinergic axis, which affects serum levels of orexins (orexin A and orexin B) [59].

The current indirect calorimetry study of T-Sham rats revealed significant increases in TEE and RQ from the level of mixed substrates used to that of greater carbohydrate utilization with an insignificant reduction in blood glucose levels (*P*=0.118). Lynn et al. [60] measured daily EE (DEE) of captive male dark-eyed juncos using a double-labeled water technique and studied the effect of subcutaneous T injection in normal males compared to controls. The results showed that elevated T levels increase some components of DEE, such as the thermic effect of locomotor activity, and lowered the contribution of others, such as resting EE. Total DEE was not higher in the T-treated males compared to that in the controls. In another investigation, Braun et al. [61] created three different androgenic levels in exercising men and found that despite the marked variation in T levels, carbohydrate oxidation, the rate of glucose disappearance, and muscular glycogen use each were highly similar across the three androgenic levels. The setting of study by Braun and colleagues was much different from the current study since we investigated resting rats. Additionally, Keller et al. [62], who investigated the relationship of androgen levels to substrate use, RQ, and EE, found that T levels do not correlate with resting EE or substrate utilization.

The effects of T on adipose tissue lipolytic enzymes may provide an explanation for the effect of T on energy homeostasis and body wt. ATGL is the initial enzyme that catalyzes TG hydrolysis into diacylglycerol (DG) inside fat droplets of adipocytes, which liberates one fatty acid. The subsequent increase in the liberation of glycerol is associated with an upregulation of the cellular enzymes essential for the oxidation of fatty acids and the release of energy [63]. The second key enzyme is HSL, which catalyzes the further hydrolysis of DG and minor quantities of TG. HSL is localized to the cytoplasm of cells, while ATGL is present on the lipid droplets and in the cytoplasm of adipocytes [64]. The current study showed increased blood levels of ATGL and reduced levels of HSL and FFA in both sham and orchiectomized rats following T administration. This effect might reduce the mass of adipose tissue and increase EE [63]. Morak et al. [65] found that a deficiency of ATGL in BAT causes a marked reduction in the expression of other esterolytic and lipolytic enzymes that are responsible for energy production. In contrast, they found that HSL deficiency causes insignificant effects. Also, a study using ATGL-knockout (AAKO) mice confirmed the potential relationship of EE with AGTL. Schoiswoh et al. [66] found that EE and VO_2_ were markedly decreased in fasted AAKO mice. Furthermore, Miyoshi et al. [49] reported that the overexpression of ATGL enzymes significantly reduces lipid droplets size and decreases TG storage, while the overexpression of HSL fails to change the size of lipid droplets or to alter TG storage in adipocytes.

Despite our findings, the current study had some limitations. The main limitation was the lack of accurate body composition analyses such as MRI to correlate our findings regarding TEE with changes in various boy compartments. Another limitation was the measurement of TEE rather than REE. Furthermore, the absence of the measurement of the luteinizing hormone and investigations of the molecular mechanisms at the intracellular level is a considerable limitation.

## 5. Conclusions

In this rat model of hypogonadism, low ghrelin/GH axis might play a role in the elevation of TEE, increased RQ, increased ATGL, hyperglycemia, low insulin, and reductions of food intake and body weight. T administration in orchiectomized rats significantly increased orexigenic mediators such as ghrelin and insulin without inducing any significant changes in wt. The mechanism for this finding might be the increase in TEE and stimulation of lipolysis through the ATGL enzyme. The associated rise of GH might help in interference with accumulation of lipid in adipose tissue. Apart from the effect on GH and HOMA-IR, T-Sham showed similar effects of T supplementation.

## Figures and Tables

**Table 1:** Mean ± SD of the biochemical parameters among studied groups (*n* = 8 per each group).

Parameters	Sham	T-Sham	Orch	T-Orch
Testosterone (ng/ml)	7.01 ± 0.91	14.95 ± 1.69	0.94 ± 0.17	4.41 ± 1.01
		*P* < 0.001^a^	*P* < 0.001^a^	*P* < 0.05^a^
				*P* < 0.001^b^

Ghrelin (pg/ml)	574.58 ± 49.11	748.26 ± 69.14	233.34 ± 38.12	705.26 ± 63.78
		*P* < 0.001^a^	*P* < 0.001^a^	*P* < 0.05^a^
				*P* < 0.001^b^

Growth hormone (pg/ml)	3671.67 ± 382.59	3824.17 ± 271.96	2356.67 ± 671.54	3318.33 ± 714.60
		*P* < 0.554^a^	*P* < 0.001^a^	*P* < 0.334^a^
				*P* < 0.001^b^

Insulin (mIU/l)	8.52 ± 0.16	9.95 ± 1.06	4.49 ± 0.74	7.86 ± 0.38
		*P* < 0.05^a^	*P* < 0.001^a^	*P* < 0.112^a^
				*P* < 0.001^b^

Glucose (mmol/l)	6.03 ± 0.28	5.63 ± 0.41	6.74 ± 0.57	5.88 ± 0.37
		*P* < 0.118^a^	*P* < 0.001^a^	*P* < 0.549^a^
				*P* < 0.05^b^

HOMA-IR	2.29 ± 0.12	2.49 ± 0.31	1.34 ± 0.24	2.05 ± 0.12
		*P* < 0.111^a^	*P* < 0.001^a^	*P* < 0.074^a^
				*P* < 0.001^b^

Cholesterol (mmol/l)	3.91 ± 0.24	8.03 ± 0.62	3.64 ± 0.79	6.92 ± 1.37
		*P* < 0.001^a^	*P* < 0.597^a^	*P* < 0.001^a^
				*P* < 0.001^b^

LDL (mmol/l)	2.29 ± 0.14	4.52 ± 0.62	1.40 ± 0.62	3.83 ± 1.12
		*P* < 0.001^a^	*P* < 0.05^a^	*P* < 0.05^a^
				*P* < 0.001^b^

HDL (mmol/l)	0.86 ± 0.08	2.01 ± 0.22	1.77 ± 0.16	1.80 ± 0.28
		*P* < 0.001^a^	*P* < 0.001^a^	*P* < 0.001^a^
				*P* < 0.756^b^

VLDL (mmol/l)	0.29 ± 0.06	0.57 ± 0.13	0.07 ± 0.01	0.48 ± 0.08
		*P* < 0.001^a^	*P* < 0.001	*P* < 0.05^a^
				*P* < 0.001^b^

TG (mmol/l)	1.44 ± 0.30	2.86 ± 0.64	0.33 ± 0.07	2.38 ± 0.38
		*P* < 0.001^a^	*P* < 0.001^a^	*P* < 0.05^a^
				*P* < 0.001^b^

HSL (pg/ml)	123.84 ± 4.67	107.38 ± 7.67	118.52 ± 3.35	64.56 ± 3.68
		*P* < 0.001^a^	*P* < 0.088^a^	*P* < 0.001^a^
				*P* < 0.001^b^

ATGL (ng/ml)	4.98 ± 0.36	23.21 ± 1.67	7.40 ± 0.53	25.18 ± 1.07
		*P* < 0.001^a^	*P* < 0.05^a^	*P* < 0.001^a^
				*P* < 0.001^b^

FFA (ng/dl)	563.52 ± 6.64	494.34 ± 3.58	569.22 ± 10.56	501.16 ± 7.06
		*P* < 0.001^a^	*P* < 0.197^a^	*P* < 0.001^a^
				*P* < 0.001^b^

Sham = normal control; T-Sham = normal rats treated with IM testosterone undecanoate preparation (100 mg/kg); Orch = orchiectomized rats; T-Orch = orchiectomized rats treated with IM testosterone undecanoate preparation (100 mg/kg); LDL = low-density lipoprotein; HDL = high-density lipoprotein; VLDL = very-low-density lipoprotein; TG = triglycerides; HSL = hormone-sensitive lipase; ATGL = adipocyte triglyceride lipase; FFA = free fatty acids. ^a^Significant versus Sham group. ^b^Significant versus Orch group.

**Table 2:** Mean ± SD of indirect calorimetry parameters of all studied groups (*n* = 8/group).

Parameters	Sham	T-Sham	Orch	T-Orch
VO_2_ (ml/h/kg)	1182 ± 61	1509 ± 113	1397 ± 61	1699 ± 94
		*P* < 0.001^a^	*P* < 0.001^a^	*P* < 0.001^a^
				*P* < 0.001^b^

VO_2_ (ml/h/rat)	456 ± 23	598 ± 44	441 ± 19	566 ± 31
		*P* < 0.001^a^	*P* < 0.001^a^	*P* < 0.001^a^
				*P* < 0.001^b^

VCO_2_ (ml/h/kg)	999 ± 50	1364 ± 116	1264 ± 60	1535 ± 55
		*P* < 0.001^a^	*P* < 0.001^a^	*P* < 0.001^a^
				*P* < 0.001^b^

VCO_2_ (ml/h/rat)	385 ± 19	541 ± 46	399 ± 19	511 ± 18
		*P* < 0.001^a^	*P* < 0.001^a^	*P* < 0.001^a^
				*P* < 0.001^b^

RQ	0.84 ± 0.01	0.90 ± 0.01	0.91 ± 0.06	0.91 ± 0.01
		*P* < 0.05^a^	*P* < 0.05^a^	*P* < 0.05^a^
				*P* < 0.001^b^

TEE (kcal/h/kg)	5.93 ± 0.18	7.62 ± 0.76	6.90 ± 0.10	8.11 ± 0.01
		*P* < 0.001^a^	*P* < 0.001^a^	*P* < 0.001^a^
				*P* < 0.001^b^

TEE (kcal/h/rat)	2.10 ± 0.35	2.28 ± 0.23	2.07 ± 0.03	2.44 ± 0.01
		*P* < 0.140^a^	*P* < 0.845^a^	*P* < 0.05^a^
				*P* < 0.05^b^

Food intake (g/rat)	38.79 ± 9.59	39.25 ± 3.65	12.98 ± 3.35	41.15 ± 14.91
		*P* < 0.933^a^	*P* < 0.001^a^	*P* < 0.662^a^
				*P* < 0.001^b^

Body weight (g)	385.50 ± 26.80	396.45 ± 43.10	315.83 ± 47.43	332.83 ± 49.49
		*P* < 0.661^a^	*P* < 0.05^a^	*P* < 0.05^a^
				*P* < 0.498^b^

Sham = normal control; T-Sham = normal rats treated with IM testosterone undecanoate preparation (100 mg/kg); Orch = orchiectomized rats; T-Orch = orchiectomized rats treated with IM testosterone undecanoate preparation (100 mg/kg); VO_2_ = volume of consumed oxygen; VCO_2_ = volume of the produced carbon dioxide; RQ = respiratory quotient (VCO_2_/VO_2_); TEE = total energy expenditure. ^a^Significant versus Sham group. ^b^Significant versus Orch group.

**Table 3:** Correlations of testosterone level with some measured parameters in all studied groups.

Parameters	Sham	T-Sham	Orch	T-Orch
Ghrelin	0.268	0.663^*∗*^	−0.754^*∗*^	−0.028
Growth hormone	0.135	0.248	0.710	0.471
Insulin	0.766^*∗*^	0.644^*∗*^	0.356	0.467
Glucose	0.613^*∗*^	0.333	−0.275	0.732^*∗*^
Cholesterol	−0.186	0.362	0.114	−0.189
Triglycerides	0.700^*∗*^	0.266	0.211	0.077
HSL	−0.356	0.040	−0.462	0.166
ATGL	0.550	−0.164	0.811^*∗*^	−0.353
FFA	−0.360	0.715^*∗*^	0.152	0.422
VO_2_	−0.193	0.150	−0.361	−0.548
VCO_2_	−0.193	0.151	0.657^*∗*^	−0.759
RQ	−0.022	0.319	0.761	0.438
TEE	0.280	0.149	0.329	0.858^*∗*^
Food intake	−0.066	0.026	0.584^*∗*^	0.023
Body weight	0.355	−0.046	0.431	0.356

Sham = normal control; T-Sham = normal rats treated with IM testosterone undecanoate preparation (100 mg/kg); Orch = orchiectomized rats; T-Orch = orchiectomized rats treated with IM testosterone undecanoate preparation (100 mg/kg); HSL = hormone-sensitive lipase; ATGL = adipocyte triglyceride lipase; FFA = free fatty acid; VO_2_ = volume of O_2_; VCO_2_ = volume of CO_2_; RQ = respiratory quotient; TEE = total energy expenditure. ^*∗*^The correlation is significant at the 0.05 level (2-tailed).

## Data Availability

The indirect calorimetry and biochemical data used to support the findings of this study are available from the corresponding author upon request.
